# Protecting intellectual property associated with health technology trials – another barrier to multi-centre trials?

**DOI:** 10.1186/1745-6215-12-S1-A52

**Published:** 2011-12-13

**Authors:** Sue Ross, Laura Magee, Stephen Wood

**Affiliations:** 1 Departments of Obstetrics and Gynaecology and Community Health Sciences, University of Calgary, Calgary, T2N 2T9, Canada; 2 Department of Medicine, University of British Columbia, Vancouver, V6H 3N1, Canada

## Objective

To examine the approaches to protection of intellectual property in multi-centre trials currently being conducted in Canada.

## Methods

Two ongoing international multicentre perinatal trials, both funded by the Canadian Institutes of Health Research, were selected for study on the basis of their contrasting approaches to protecting intellectual property. These approaches were examined in detail to understand their motivation, and to estimate the impact of these approaches on centre recruitment.

## Results

**CHIPS (Control of Hypertension in Pregnancy Study, ISRCTN71416914)** – is recruiting 1028 pregnant women in 14 countries. Women with hypertension are randomised to tight or less tight control of hypertension. Primary outcome: composite of pregnancy loss/neonatal intensive care.

Intellectual property is safeguarded by publishing the protocol online [1]. Positive consequences: possible/actual sites have easy access to full study design; potential for open discussion between collaborators; study investigators will be held to high standards of reporting. Negative consequences: details are available with potential for plagiarism.

**FACT (Folic Acid Clinical Trial, ISRCTN23781770)** – is recruiting 3656 pregnant women in 4 countries. Pregnant women are randomised to receive either 4 mg folic acid or placebo daily. Primary outcome: development of pre-eclampsia.

Intellectual property is safeguarded by requiring local investigators/institutions to sign non-disclosure agreements (NDAs) before the full protocol is provided. Positive consequences: details of the study not available unless legal agreement is signed. Negative consequences: may restrict academic openness; provide additional barriers to site recruitment; investigators may present selected results.

## Conclusions

The two trials illustrate contrasting approaches to protecting intellectual property associated with study design. This issue is becoming more important for academic institutions whose reputations and wealth are influenced by ownership and management of the intellectual property generated by faculty members. Some institutions prefer to manage risk using legal measures. In the case of trials, institutions protect their intellectual property by introducing NDAs into the sub-site agreement process.

NDAs between the lead institution and sub-sites may represent a legally responsible approach. Unfortunately there are potential disadvantages: adding an extra legal step into sub-site recruitment will make this process more difficult; this step may reduce academic openness and collegiality; and restricting the availability of the protocol could allow investigators to present selected results.

The use of non-disclosure agreements is an increasing trend in Canada. This trend will impact on the work of clinical trialists, perhaps making site recruitment even more difficult.
